# The simulacra of shame: the complexity of trauma in adoptive parenthood of second generation

**DOI:** 10.3402/ejpt.v4i0.22981

**Published:** 2013-12-05

## XIII ESTSS Conference: “Trauma and its clinical pathways: PTSD and beyond”, Bologna, June 2013 ORAL, JUNE 8 HALL SYDNEY

## Contesti traumatici complessi

### I simulacri della vergogna: la complessità del trauma adottivo nella genitorialità di seconda generazione   15.15–15.30

La sofferenza silenziosa dell'abbandono precoce non elaborata scava rumorosamente anche nell'attitudine genitoriale soprattutto nella possibilità adottiva. Figli adottivi che creano a loro volta una famiglia di accoglienza adottiva trovano un potenziale traumatogeno pericoloso nel vivere dalla parte genitoriale una esperienza filiale conosciuta. A volte queste strutture familiari, che non sono riuscite a delimitare i perimetri di ruolo e che dovrebbero fornire elementi capaci di dare significato all'esperienza, saltano scegliendo come unico linguaggio praticabile il sintomo. Il sintomo diventa quindi manifesto di ciò che viene celato nell'incapacità di adeguare le risorse psicoaffettive genitoriali alle esigenze esplorative, conoscitive ed integrative di figli che hanno vissuto a loro volta l'abbandono. I figli diventano inconsapevoli simulacri dell'altrui vergogna, messa a nudo solo nel rispecchiamento filiale al quale viene consegnata in tutta la sua inelaborabilità. Il potere destrutturante di ciò che non è stato affrontato mette infatti a nudo l'inermità e la vergogna che continua l'opera disorganizzante spingendo il soggetto verso autodistruttività e nascondimento. E quando l'esperienza traumatica ha avuto la meglio sulle risorse individuali ed ambientali lascia nel proprio bagaglio strumenti non efficaci che alterano la capacità di lettura di sé e del mondo, scoprendo improvvisamente la muta disperazione di questi bambini lasciati soli di fronte al proprio trauma che trasforma *autoplasticamente* la propria psiche rinunciando temporaneamente a cercare di modificare *alloplasticamente* il mondo esterno in quanto non ancora conosciuto. Dalla parte genitoriale, la vergogna vela profondi sentimenti di annichilimento, impotenza e conseguente passività in quanto non sempre i consueti schemi difensivi possono mantenersi efficaci e la regressione diventa una risposta massiva all'imponente senso di irrealtà e sospensione traumatica.

Ardino V. (Ed.) (2011), *Posttraumatic Syndromes in Childhood and Adolescence*. Wiley-Blackwell.

Caretti V., Schimmenti A. (2007), *Prefazione. Il fallimento delle relazioni primarie e il trauma evolutivo*. In: Bifulco A., Moran P. (1998), *II Bambino Maltrattato*. Casa Editrice Astrolabio, Roma.

Ferruta A. (2004), *Esperienza clinica e ricerca empirica a confronto*. In: Alberto G.G. (Ed), *Il cambiamento nella psicoterapia di crisi*. Franco Angeli, Milano.

### The simulacra of shame: the complexity of trauma in adoptive parenthood of second generation

#### Elena Acquarini Dipartimento di Scienze dell'Uomo, Università degli Studi di Urbino, Urbino, Italy

The silent suffering of the early and unprocessed abandon digs noisily even in the parental attitude, especially in adoptive possibility. Adopted children who in turn create a foster family found a potential traumatogenic dangerous living *on the side* of the parental adoptive child. Sometimes, these family structures, which have failed to define the perimeters of roles and that should provide elements to be able to give meaning to child experience, jump by choosing as the sole-language practicable symptom. Children become unwitting simulacra of the other person's shame, bare only in reflective-mode branch which is delivered in its entire unprocessing mode. The destructive power of what has not been addressed puts it bare helplessness and shame that continues its disorganizing work pushing the subject towards self-destruction and concealment. If so, when the traumatic experience had its best on individual resources and environmental, leaves in your baggage tools not effective that alter the reading ability of self and the world, discovering suddenly the wetsuit despair of these children. The trauma of orphan children transforms autoplastically and their own psyche temporarily renouncing to attempt to modify alloplastically the outside world as not yet known. From the parental attitude, shame hides deep sentiments of annihilation, impotence, and consequent liabilities for the usual defensive schemes cannot remain effective and the regression becomes a massive response to an impressive sense of unreality and a traumatic suspension.

